# Effects of fatigue on the *in vivo* kinematics and kinetics of talocrural and subtalar joint during landing

**DOI:** 10.3389/fbioe.2023.1252044

**Published:** 2023-09-08

**Authors:** Ye Luo, Zhuman Li, Mengling Hu, Ling Zhang, Feng Li, Shaobai Wang

**Affiliations:** ^1^ School of Exercise and Health, Shanghai University of Sport, Shanghai, China; ^2^ Key Laboratory of Exercise and Health Sciences of Ministry of Education, Shanghai University of Sport, Shanghai, China

**Keywords:** dual fluoroscopic imaging system, fatigue, talocrural joint, subtalar joint, *in vivo* kinematics

## Abstract

**Objective:** Fatigue can affect the ankle kinematic characteristics of landing movements. Traditional marker-based motion capture techniques have difficulty in accurately obtaining the kinematics of the talocrural and subtalar joints. This study aimed to investigate the effects of fatigue on the talocrural and subtalar joints during the landing using dual fluoroscopic imaging system (DFIS).

**Methods:** This study included fourteen healthy participants. The foot of each participant was scanned using magnetic resonance imaging to create 3D models. High-speed DFIS was used to capture images of the ankle joint during participants performing a single-leg landing jump from a height of 40 cm. Fatigue was induced by running and fluoroscopic images were captured before and after fatigue. Kinematic data were obtained by 3D/2D registration in virtual environment software. The joint kinematics in six degrees of freedom and range of motion (ROM) were compared between the unfatigued and fatigued conditions.

**Results:** During landing, after the initial contact with the ground, the main movement of the talocrural joint is extension and abduction, while the subtalar joint mainly performs extension, eversion, and abduction. Compared to unfatigued, during fatigue the maximum medial translation (1.35 ± 0.45 mm vs. 1.86 ± 0.69 mm, *p* = 0.032) and medial-lateral ROM (3.19 ± 0.60 mm vs. 3.89 ± 0.96 mm, *p* = 0.029) of the talocrural joint significantly increased, the maximum flexion angle (0.83 ± 1.24° vs. 2.11 ± 1.80°, *p* = 0.037) of the subtalar joint significantly increased, and the flexion-extension ROM (6.17 ± 2.21° vs. 7.97 ± 2.52°, *p* = 0.043) of the subtalar joint significantly increased.

**Conclusion:** This study contributes to the quantitative understanding of the normal function of the talocrural and subtalar joints during high-demand activities. During landing, the main movement of the talocrural joint is extension and abduction, while the subtalar joint mainly performs extension, eversion, and abduction. Under fatigue conditions, the partial ROM of the talocrural and subtalar joints increases.

## 1 Introduction

Landing is a common movement in many sports and military training and places high demands on the joints and tissues of the lower limbs (Sell et al., 2010). During landing impact, the vertical ground reaction force (vGRF) can easily reach several times the body weight (BW) (Niu et al., 2014), making it one of the most challenging and high-risk movements (Knapik et al., 2004; Sell et al., 2010).

Previous studies suggest that the knee joint and related muscle tissues may be the main contributors to energy absorption during landing (Zhang et al., 2000; Tamura et al., 2021). However, the contribution of each joint to energy dissipation may be influenced by various factors, such as landing strategy and fatigue (Heebner et al., 2017). During a soft landing, the hip and knee joints are the main contributors to energy absorption (Devita and Skelly, 1992). During a hard landing, the ankle joint is the primary contributor to energy absorption (Self and Paine, 2001; Heebner et al., 2017). Moreover, a fatigued landing exhibited similar results, with the lower limbs adopting a stiffer posture (with more knee extension) during landing to cope with the impact (Brazen et al., 2010; Santamaria and Webster, 2010; Weinhandl et al., 2011). Additionally, the contribution of the ankle joint to energy absorption during the landing impact phase has been shown to increase after fatigue (Orishimo and Kremenic, 2006; Weinhandl et al., 2011). These findings suggest that, as fatigue or exercise intensity increases, individuals tend to use a landing strategy dominated by the ankle joint (Zhang et al., 2021). Under fatigue conditions, there is an increased load transfer to the ankle joint, leading to a greater absorption of impact energy by the ankle joint (Orishimo and Kremenic, 2006; Weinhandl et al., 2011; Zhang et al., 2021).

Fatigue is a common phenomenon occurring after high-intensity or prolonged exercise. The effects of fatigue on landing have been extensively investigated in biomechanical studies (Santamaria and Webster, 2010; Tamura et al., 2016). However, traditional marker-based motion capture techniques often model the complicated foot and ankle complex as a single segment (Schmitz et al., 2007; Okita et al., 2009), ignoring the movement between different bones (Pothrat et al., 2015; Ridder et al., 2015). This method is also affected by the relative movement of the markers and bone, which can lead to soft tissue artifacts (STA) (Schallig et al., 2021). Additionally, wearing shoes affects accuracy during capture (Arndt et al., 2013), and there is a lack of palpable bony landmarks on the talus (Roach et al., 2021). Although the use of intracortical pins can eliminate the effects of the STAs, they are invasive and inapplicable to high-speed actions (Arndt et al., 2013). To date, no studies have reported on the kinematics of the talocrural and subtalar joints that form the ankle joint during single-leg landing jump movements. The dual fluoroscopic imaging system (DFIS) is a method for tracking ankle joint kinematics *in vivo* (Neubert et al., 2017; Pitcairn et al., 2020). During dynamic movements such as jumping, the system exhibited capturing translational errors of 0.5 ± 0.2 mm and 0.8 ± 0.5 mm for the talocrural and subtalar joints, respectively (Pitcairn et al., 2020). The rotational errors for the talocrural and subtalar joints were 1.4° ± 0.4° and 1.5° ± 0.5°, respectively (Pitcairn et al., 2020). Using DFIS to further understand the kinematics of the talocrural and subtalar joints during landing and the effects of fatigue. This will deepen our understanding of the kinematics of the talocrural and subtalar joints.

The aim of this study is two-fold: First, we aim to investigate the kinematics of the talocrural and subtalar joint during single-leg landing jump using DFIS. Second, we aim to investigate the effects of fatigue on the kinematics of the talocrural and subtalar joint during single-leg landing jump. We hypothesized that fatigue increases the angle and translation ROM of the ankle.

## 2 Materials and methods

### 2.1 Participants

Fourteen healthy male participants (age: 21.6 ± 1.3 years, height: 176.9 ± 4.1 cm, weight: 69.91 ± 5.5 kg) were recruited through poster advertisements. All the participants provided informed consent. The study protocol was approved by the institutional ethics committee (No. 102772021RT133). The sample size was determined using G*Power software (Version 3.1.9.7, Kiel University, Kiel, Germany) and the required minimum sample size was calculated to be 14 participants to achieve a power of 0.8 (significance level α: 0.05) (McHenry et al., 2015). All participants regularly performed moderate-intensity exercise (two to four times/week, 30 min+/session). None of the participants had a history of lower limb injury.

### 2.2 3D model reconstruction

**FIGURE 1:**
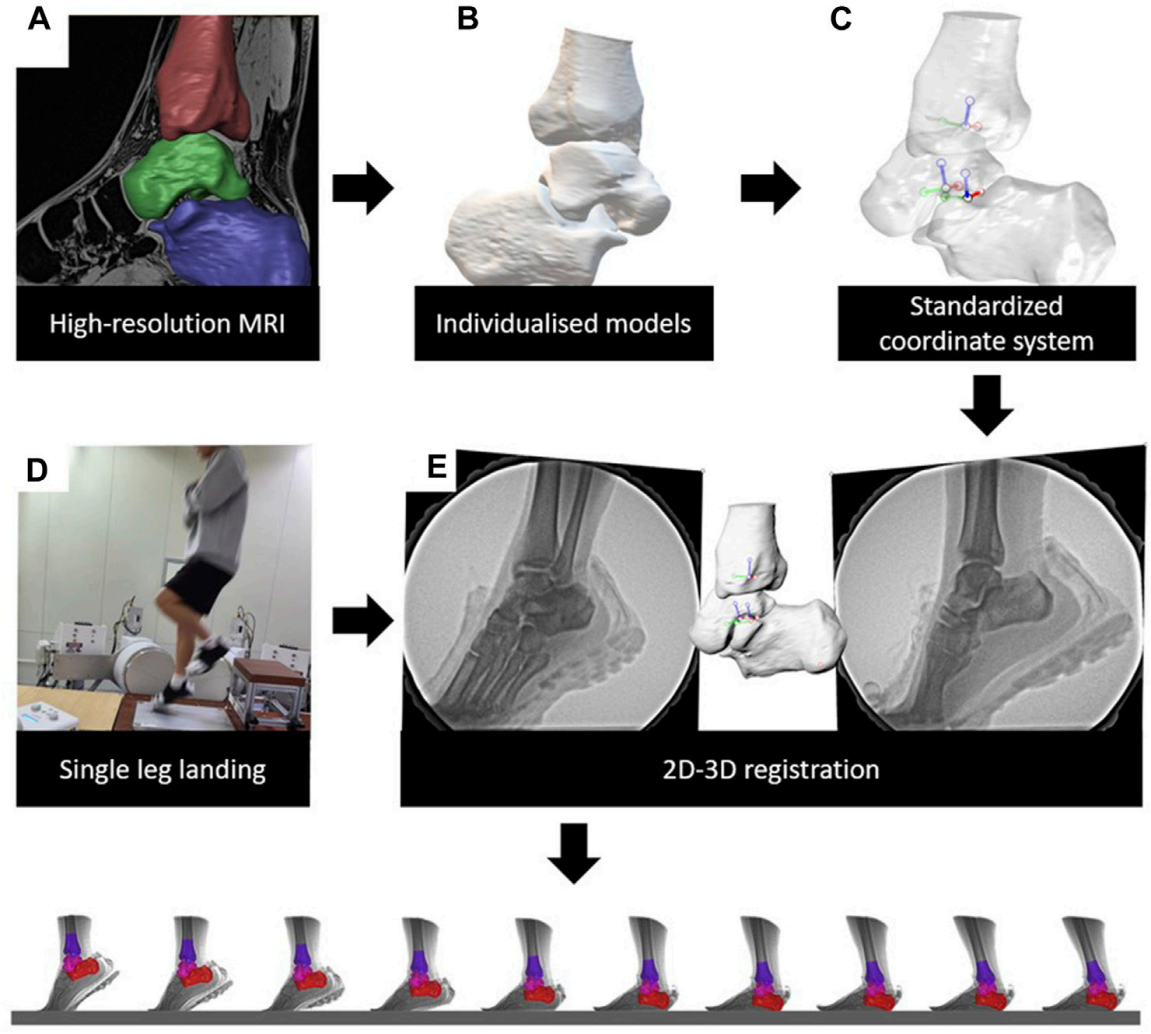
Data collection and process of DFIS. **(A)** High resolution MRIs were collected. **(B)** Acquisition of individualized 3D models. **(C)** Creation of coordinate systems for each model. **(D)** Acquisition of high-speed dual fluoroscopic imaging system data. **(E)** Combine MRI and DFIS data to acquire the kinematics.

Each participant underwent a 3.0T magnetic resonance imaging (MRI) (MAGNETOM Prisma; Siemens Healthcare, Erlangen, Germany) scan of their ankles. MRI is a promising radiation-free alternative to computed tomography (CT) for providing CT-like visualization of bones (Florkow et al., 2022). The procedure was set as a T1-weighted three-dimensional sequence with phase-encoding gradient echo (resolution: 0.6 × 0.6 × 0.6 mm; flip angle: 10°; repetition time: 10.5 ms; echo time: 4.92 ms). The participants laid supine with the ankle joint in a relaxed neutral position. MRI data were used to establish 3D ankle joint models. Images of the calcaneus, talus, and tibia were segmented using 3D-reconstruction software (Amira 3D 2021.2, Thermo Fisher Scientific, Waltham, MA, USA) to create 3D models (Figures 1A, B).

### 2.3 Coordinate system establishment

Anatomic coordinate systems of the models were created by the same skilled researchers based on a previously described method (Figure 1C) (Yamaguchi et al., 2009; Ye et al., 2023).

Tibia: The origin of the coordinate system is at the point where the long axis of the distal tibial shaft crosses the tibial plateau. The long axis of the tibial shaft was defined as the superior-inferior axis. The long axis is fitted to a straight line through the centers of multiple parallel cross-sections. The anteroposterior axis passes through the origin and is perpendicular to the line connecting the anteromedial and the anterolateral edges of the tibial plafond. The mediolateral axis was defined as the line perpendicular to the anterior-posterior and superior-inferior axes.

Talus: The midpoints of the anterior medial and anterior lateral edges and the midpoints of the posterior medial and posterior lateral edges of the trochlea tali were determined. A circle was drawn such that it contained two points, and the arc approximated the trochlea. The origin of the coordinate system is at the center of the circle. The mediolateral axis is defined as the line perpendicular to the circle passing through the origin. The anterior-posterior axis was defined as a line perpendicular to the mediolateral and superior-inferior axes.

Calcaneus: The origin of the coordinate system was the midpoint of the line connecting the most lateral point of the posterior articular surface to the most medial point of the middle articular surface. The superior-inferior axis was defined as the line perpendicular to the line connecting these two points and perpendicular to the inferior surface of the calcaneus. The medial-lateral axis was defined as the line perpendicular to the superior-inferior axis and lateral wall of the calcaneus. The anterior-posterior axis was defined as a line perpendicular to the mediolateral and superior-inferior axes.

### 2.4 High-speed fluoroscopy setup and fatigue protocols

After the MRI scans, the participant underwent imaging using the DFIS (Figure 1D). The DFIS consisted of two sets of X-ray emitters and image intensifiers. First, the positions of the X-ray emitters and image intensifiers were adjusted to ensure a complete capture of the landing phase during a single-leg jump, and calibration images were obtained. These images were used to correct for intrinsic distortion in the captured images. The included angle of the two sets of fluoroscopy systems was 120° and the source to image distance was 130 cm. The energy settings of the DFIS were as follows: a voltage of 55 kV, current of 50mA, sampling frequency of 250fps, exposure time of 700 μs, and image resolution of 1,024 × 1,024 pixels. The system was synchronized with a force plate (Kistler 9286BA; Kistler Corporation, Winterthur, Switzerland) at a sampling frequency of 1,000 Hz.

During the experiment, the participants were asked to wear standard lab-supplied experimental vests, shorts, and shoes (traditional running footwear, 3515 WM-053, midsole material: EVA, TPU; heel-to-toe drop, 5 mm; upper structure, textile fabric; without any arch support). All participants performed a 5-min running warm-up before formal data collection. To improve the rigor and consistency of the experiment, all participants were asked to stand on a 40 cm high platform with their arms crossed and to complete a single-leg landing-jump movement. The participants landed on the force plate with their dominant foot (as determined using a preferred kicking foot questionnaire). The participants were asked not to jump off or lower their center of mass prematurely. Landing practice sessions were conducted before formal data collection to ensure that the participants could perform the task correctly. To reduce the amount of radiation exposure to the participants, only one successful experiment before and after fatigue for each participant was collected and analyzed, guided by the quality of the X-ray images. The single collection time is about 0.6s. The total radiation associated with DFIS in this study was calculated to be estimated at 0.8 mSv. This radiation exposure is well below the annual occupational limit of 50 mSv systemic effective dose limit established by the U.S. Nuclear Regulatory Commission (Shore, 2014; Cross et al., 2017).

Fatigue was induced by wearing a weight vest (16 kg) and running for 3 km at a speed of 12 km/h. The termination criteria were completion of the 3 km run with a Borg scale rating greater than 17 (very hard) or inability to continue the run with a Borg scale rating greater than 19 (very, very hard) (Tamura et al., 2016). A Borg scale poster was placed in front of the treadmill and the research assistant asked about the scale every 500 m.

### 2.5 Data processing

**FIGURE 2:**
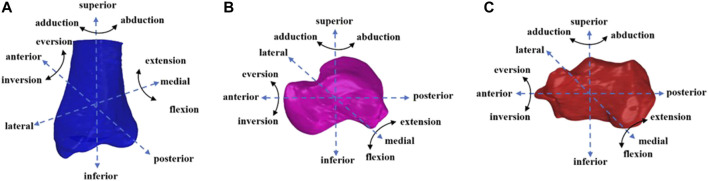
**(A)** Tibia, **(B)** talus, **(C)** calcaneus motin diagram.

After collecting the data, the 3D bone model and calibrated X-ray images were imported into a 3D virtual environment simulation software (Rhinoceros 7.0, McNeel and Associates, Seattle, United States). We conducted 3D/2D registration and the talocrural and subtalar joints kinematics were calculated (Figure 1E). In this study, the ankle position obtained during the standing position was used as the zero-reference position to obtain “relative” kinematic data. The kinematic data for the corresponding hindfoot landing task were obtained by subtracting the zero-reference position. The kinematics within 100 ms of foot contact (Myers et al., 2011), which was the most critical buffering stage during landing, were analyzed (Devita and Skelly, 1992; Schot et al., 1994; Norcross et al., 2013). The “foot contact” is defined as the moment when the vGRF is greater than 20N (Harry et al., 2019). The force measured during landing was standardized based on the participant’s body weight (BW). Kinematic data were presented in the form of six degrees of freedom (6DOF) (Figure 2), including translation in the lateral/medial, anterior/posterior, and superior/inferior directions, as well as flexion/extension, eversion/inversion, and abduction/adduction. Positive values indicate medial translation, posterior translation, inferior translation, flexion, eversion, and abduction of the talus relative to the tibia (calcaneus relative to the talus), whereas negative values indicate the opposite.

### 2.6 Statistics

The mean and standard deviation were calculated for each kinematic and kinetic variable, respectively. Shapiro–Wilk tests were used to assess the normality of the variables. SPSS v27.0 (IBM Corp., Armonk, NY, USA) was used for all statistical analyses. After confirming homogeneity of variance, one-way repeated measures ANOVA were performed to determine the differences in 6DOF between the unfatigued and fatigued conditions. The significance level was set at *p* < 0.05.

## 3 Results

### 3.1 *In vivo* kinematics of the talocrural and subtalar joints

In this study, all participants exhibited a forefoot landing strategy during the first landing phase of single-leg landing jump.

#### 3.1.1 Talocrural joint

**FIGURE 3:**
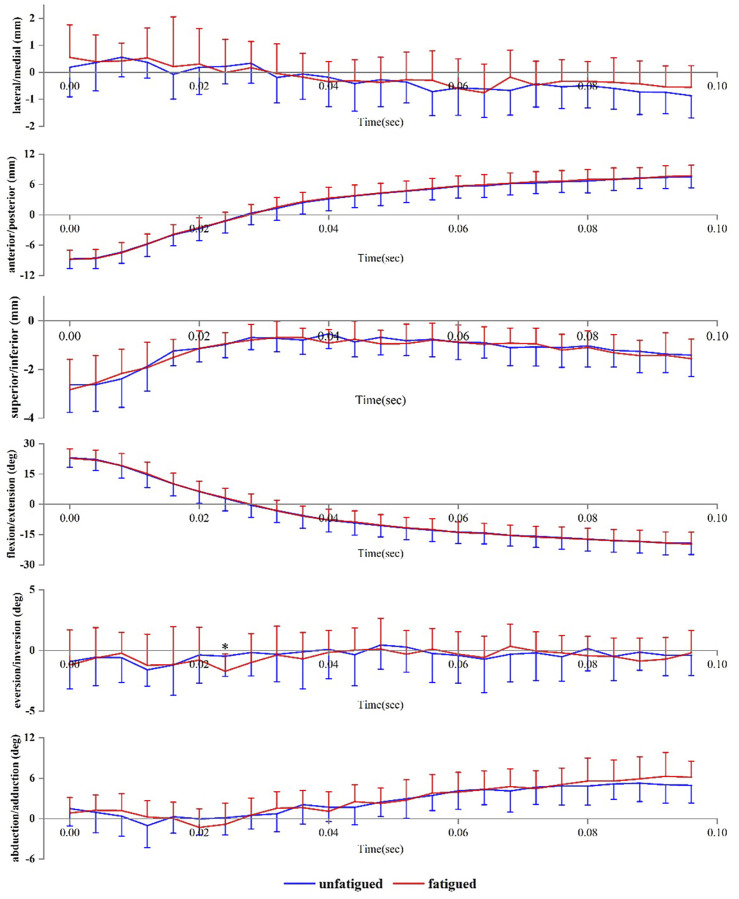
Tibiotalar joint positions during landing. Data are presented as mean values with standard deviations. Positive values indicate medial translation, posterior translation, inferior translation, flexion, eversion, and abduction, while negative values indicate the opposite. *, significant difference between unfatigued and fatigued (*p* < 0.05).

Figure 3 presents the angle and translation changes of the talocrural joint during the landing phase in both the unfatigued and fatigued conditions. For the talocrural joint, the motion after initial contact was extension and abduction, whereas the translational movements were lateral, posterior, and inferior translation. Compared to unfatigued conditions, fatigued conditions showed that the talocrural joint inverted more at 24 ms after initial contact (unfatigued vs. fatigued, −0.48 ± 1.67° vs. −1.72 ± 1.42°, *p* = 0.043).

#### 3.1.2 Subtalar joint

**FIGURE 4:**
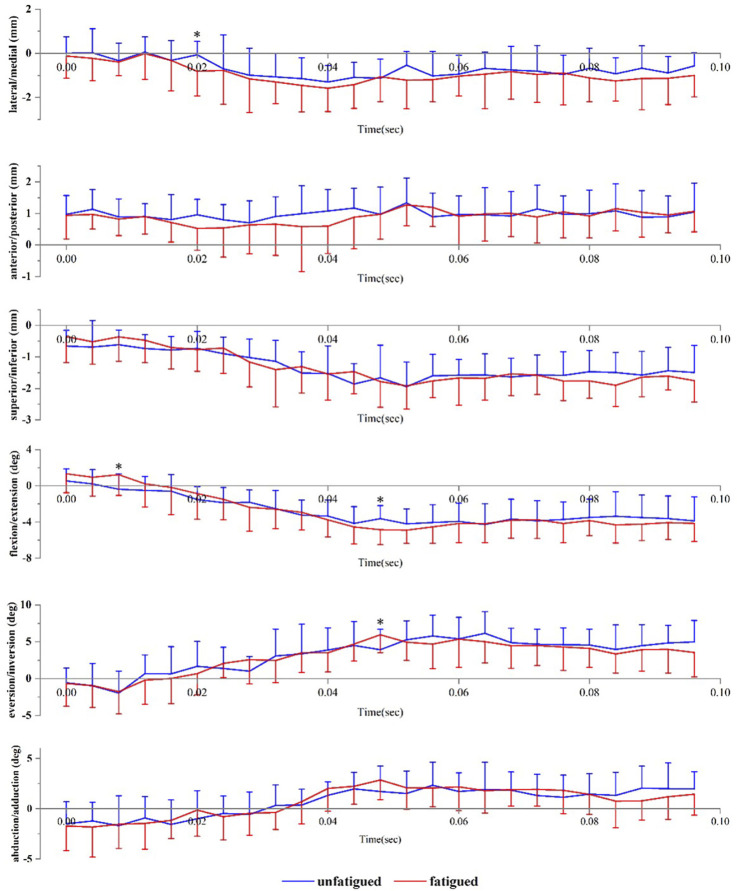
Subtalar joint positions during landing. Data are presented as mean values with standard deviations. Positive values indicate medial translation, posterior translation, inferior translation, flexion, eversion, and abduction, while negative values indicate the opposite. *, significant difference between unfatigued and fatigued (*p* < 0.05).

Figure 4 presents the angle and translation changes of the subtalar joint during the landing phase in both the unfatigued and fatigued conditions. For the subtalar joint, the motion after initial contact was extension, eversion, and abduction. The translational movements were superior and lateral translation. Data are presented as unfatigued versus fatigued data. Compared to unfatigued conditions, in fatigued conditions, the talocrural joint had more flexion (−0.39 ± 1.72° vs. 1.22 ± 2.28°, *p* = 0.044) at 8 ms after initial contact, more medial translation (−0.06 ± 0.60 mm vs. −0.81 ± 1.11 mm, *p* = 0.035) at 20 ms after initial contact, and more extension (−3.63 ± 1.45° vs. −4.86 ± 1.65°, *p* = 0.047) and eversion (3.91 ± 2.77° vs. 5.94 ± 2.43°, *p* = 0.049) at 48 ms after initial contact.

### 3.2 Peak translation, angles, and range of motions of the talocrural and subtalar joints

#### 3.2.1 Talocrural joint

**FIGURE 5:**
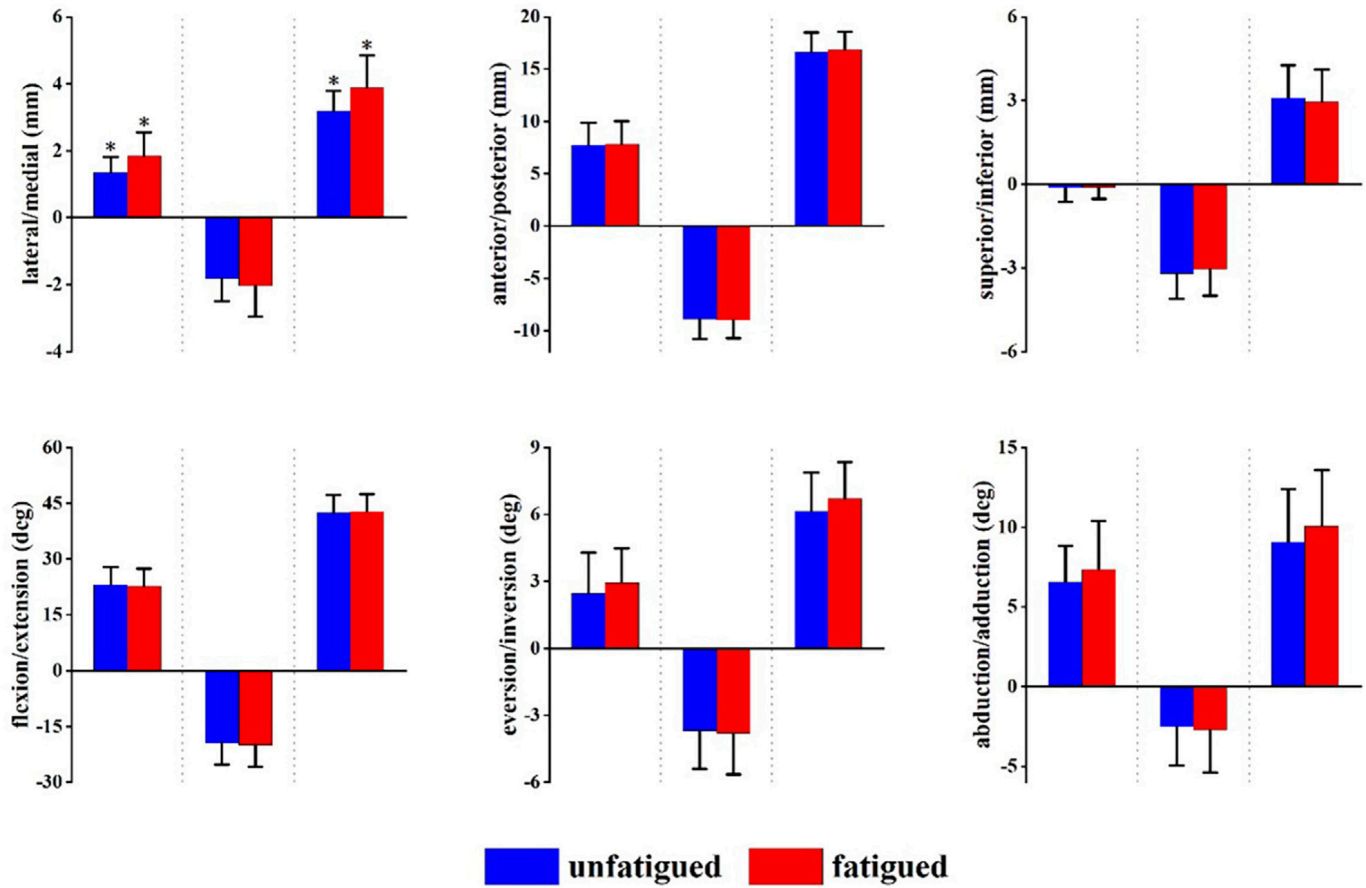
Peak translation and rotation of the talocrural joint in unfatigued and fatigued conditions. From left to right each translation and rotation direction in order of maximum, minimum, and range of motion (ROM). Positive values indicate medial translation, posterior translation, inferior translation, flexion, eversion, and abduction, while negative values indicate the opposite. *p < 0.05, significant difference between unfatigued and fatigued.

The data are presented as unfatigued versus fatigued data. Compared to unfatigued conditions, fatigued conditions showed an increase in the maximum medial translation of the tibiotalar joint (1.35 ± 0.45 mm vs. 1.86 ± 0.69 mm, *p* = 0.032), and medial-lateral range of motion (ROM) (3.19 ± 0.60 mm vs. 3.89 ± 0.96 mm, *p* = 0.029) (Figure 5).

#### 3.2.2 Subtalar joint

**FIGURE 6:**
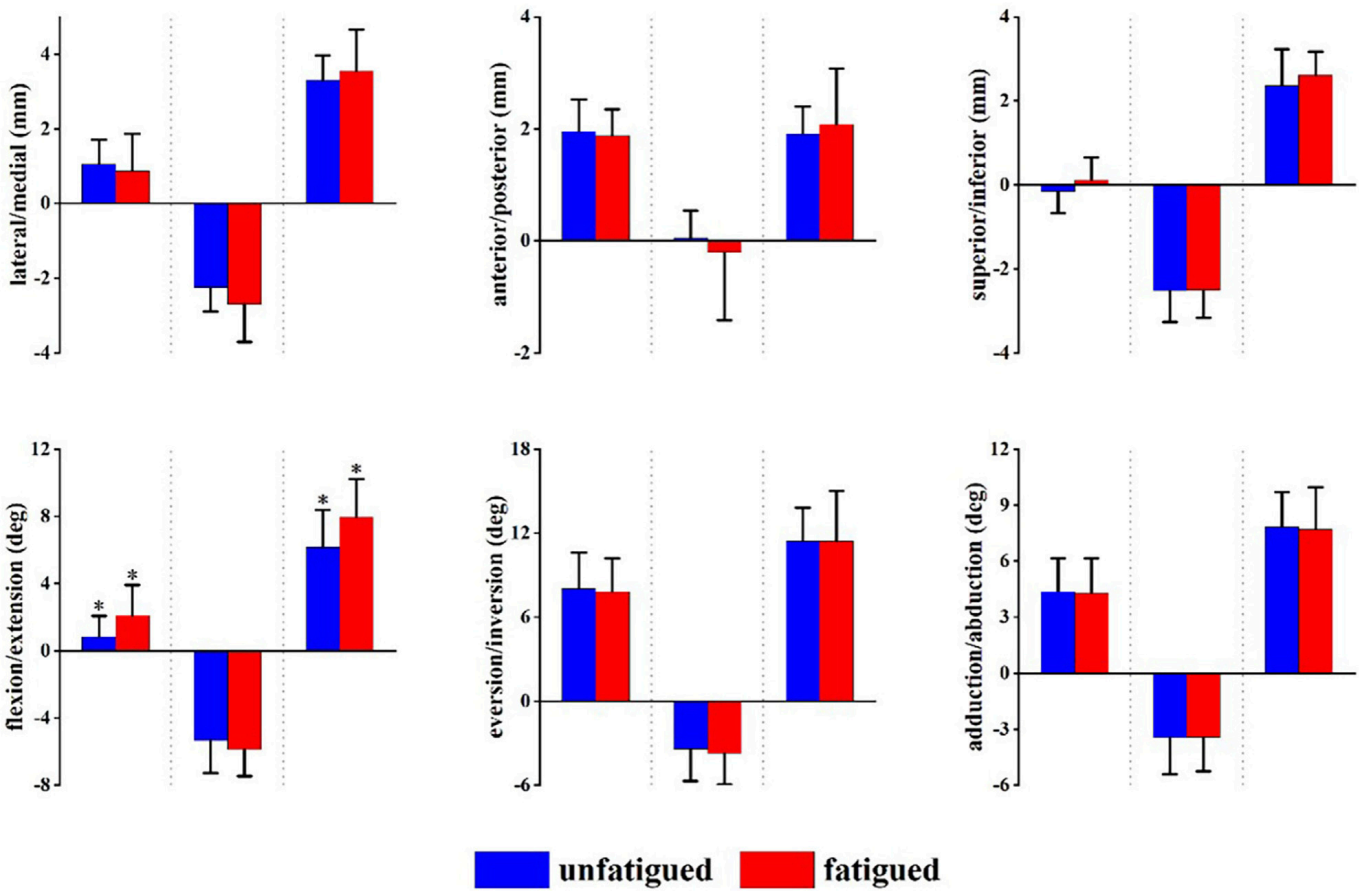
Peak translation and rotation of the subtalar joint in unfatigued and fatigued conditions. From left to right each translation and rotation direction in order of maximum, minimum, and range of motion (ROM). Positive values indicate medial translation, posterior translation, inferior translation, flexion, eversion, and abduction, while negative values indicate the opposite. *p < 0.05, significant difference between unfatigued and fatigued.

The data are presented as unfatigued versus fatigued data. Compared to unfatigued conditions, fatigued conditions showed an increase in the maximum flexion angle of the talocrural joint (0.83 ± 1.24° vs. 2.11 ± 1.80°, *p* = 0.037) and flexion-extension ROM (6.17 ± 2.21° vs. 7.97 ± 2.52°, *p* = 0.043) (Figure 6).

### 3.3 Peak vertical ground reaction force

The data are presented as unfatigued versus fatigued data. Compared to unfatigued conditions, fatigued conditions showed the time to reach peak vertical ground reaction force (43 ± 6 ms vs. 43 ± 5 ms, *p* = 0.894) and vGRF (2977.73 ± 450.10 N vs. 3106.64 ± 500.46 N, 3.54 ± 0.54 BW vs. 3.70 ± 0.60 BW, *p* = 0.480) was not affected.

## 4 Discussion

In this study, we report the *in vivo* kinematics of the talocrural and subtalar joints during the landing phase of a single-leg jump using a combination of MRI and DFIS. We investigated the effects of fatigue on the talocrural and subtalar joints. This study found an increase in the maximum flexion angle and flexion-extension ROM of the subtalar joint under fatigued conditions. Maximum medial translation and mediolateral ROM of the talocrural joint also increased. These results are consistent with our hypotheses.

Our study found that the main rotational motion of the talocrural joint after landing is extension with abduction. This is consistent with a previous *in vitro* study that reported the extension and abduction of the ankle joint under load-bearing conditions (Ito et al., 2017). Ankle extension could disperse the high vGRF received by the foot during landing, and abduction may be more beneficial for load bearing of the ankle (Ito et al., 2017). However, our results are somewhat different from the results of Fukano et al.'s study (Fukano et al., 2014), which did not find abduction of the talocrural joint during landing. This can be explained by the lower landing height (10 cm) used in their experiment (Fukano et al., 2014). This could also be due to differences in equipment. A single-plane fluoroscopic equipment was used in the study of Fukano et al.'s study (Fukano et al., 2014). The 3D/2D registration technology based on a single-plane may bring greater uncertainty in capturing movements outside the plane (Tsai et al., 2006). The translational motion of the ankle joint during landing mainly moves laterally, posteriorly, and inferiorly, which assists in extension.

The rotational motions of the subtalar joint during landing include flexion, eversion, and abduction. This finding is also consistent with that of a previous study (Fukano et al., 2014). We found that eversion and abduction of the subtalar joint started only after contact of the metatarsal with the ground. Moreover, there was a tendency towards superior and lateral translation. This additional information may be due to the differences in movement or the use of equipment with a higher sampling frequency (250 Hz). The use of high sampling frequency enables the presentation of more detailed and accurate *in vivo* movement information for high-speed movements. The translation of this joint was small (<5 mm) during translational movements, which was likely because of the tight soft-tissue structures around the joint (Sangeorzan and Sangeorzan, 2018).

In the fatigued condition, the maximum medial translation and medial-lateral ROM of the talocrural joint increased, and the maximum flexion angle and flexion-extension ROM of the subtalar joint increased. This may have been due to muscle fatigue. Muscles constitute the dynamic stabilizers of the joint. Following the fatigue-inducing process, the strength of the muscles decreases, leading to a reduced ability to maintain dynamic stability. In the condition of muscle fatigue, joints rely more on bone structure and ligaments to maintain stability, which result in increased joint mobility. Anatomically, the talocrural joint is fixed in the joint socket formed by the tibia and fibula. The movement of the talocrural joint in the sagittal plane is associated with the tibiofibular joint. The stability of the tibiofibular joint is maintained by the strong syndesmotic ligament, which resists forces that separate the two bones (Ebraheim et al., 2006). Therefore, the constraints imposed by the bones and ligaments limit the medial-lateral translation of the talocrural joint during movement, and the increased ROM observed after fatigue is also limited. In contrast, the stability of the subtalar joint is primarily provided by the joint capsule and ligaments surrounding the joint. Therefore, in a fatigued state, the increase in range of motion of the subtalar joint is greater compared to the talocrural joint.

Our study has the following limitations: First, the lack of female participants included in the study reduced the generalizability of the results to females. However, this also avoids the potential effects of sex differences. Therefore, the effects of sex differences should be explored in future studies. Second, the knee and hip joints simultaneously influence the force absorption of the movement during landing. However, owing to equipment limitations, the knee and hip joints cannot be analyzed simultaneously with the ankle joint during landing. We will improve the equipment or research protocol to allow for the synchronous analysis of the knee and ankle joints during landing. Lastly, participants’ movements may vary significantly under expected and unexpected conditions. This study and most motion capture experiments aimed to ensure rigor by using standardized postures (crossed arms, same landing height, etc.). However, this standardization may reduce the generalizability of the data under unexpected conditions. Nevertheless, the current study effectively reflects the *in vivo* kinematics of the talocrural and subtalar joints during expected landing.

## 5 Conclusion

This study contributes to quantitatively understanding the normal function of the talocrural and subtalar joints in healthy males during landing tasks under both unfatigued and fatigued conditions. During landing, the talocrural joint movements are extension and adduction, while the translational movements are lateral, posterior, and inferior. For the subtalar joint, the motion after initial contact was extension, eversion, and abduction. The findings of this study indicate an increase in partial ROM of the talocrural and subtalar joints under fatigue conditions.

## Data Availability

The raw data supporting the conclusion of this article will be made available by the authors, without undue reservation.
